# Profile and Determinants for Complications of Imported Malaria in 5 Chinese Provinces From 2014 to 2021: Retrospective Analysis

**DOI:** 10.2196/52089

**Published:** 2024-08-30

**Authors:** Bowen Liu, Tao Zhang, Duoquan Wang, Shang Xia, Weidong Li, Xiaoxi Zhang, Shuxun Wang, Xiao-Kui Guo, Xiao-Nong Zhou, Shizhu Li

**Affiliations:** 1 School of Global Health, Chinese Center for Tropical Diseases Research, Shanghai Jiao Tong University School of Medicine, Shanghai, China; 2 One Health Center, Shanghai Jiao Tong University-The University of Edinburgh, Shanghai, China; 3 Anhui Provincial Center for Disease Control and Prevention, Hefei, Anhui Province, China; 4 National Key Laboratory of Intelligent Tracking and Forecasting for Infectious Diseases, National Institute of Parasitic Diseases at Chinese Center for Disease Control and Prevention, Chinese Center for Tropical Diseases Research, Shanghai, China; 5 NHC Key Laboratory of Parasite and Vector Biology, Shanghai, China; 6 WHO Collaborating Center for Tropical Diseases, National Center for International Research on Tropical Diseases, Shanghai, China

**Keywords:** imported malaria, epidemiological characteristics, complications, influencing factors, China

## Abstract

**Background:**

In 2021, the World Health Organization officially declared the People’s Republic of China as malaria-free. However, despite this milestone achievement, the continued occurrence of severe and fatal cases of imported malaria in China, due to globalization and increased international communication, remains a significant public health concern.

**Objective:**

The aim of this study was to elucidate the epidemiological characteristics of imported malaria in 5 Chinese provinces from 2014 to 2021 and to identify the factors that influence complications in imported malaria cases. The findings will provide a basis for enhancing prevention and control measures, thereby consolidating China’s achievements in malaria elimination.

**Methods:**

A case-based retrospective study was performed, using surveillance data collected from the representative provinces of China from 2014 to 2021. Epidemiological characteristics were analyzed using descriptive statistics. Logistic regression was used to identify the factors influencing the occurrence of complications.

**Results:**

A total of 5559 malaria cases were included during the study period. The predominant species was *Plasmodium falciparum* (3940/5559, 70.9%), followed by *Plasmodium ovale* (1054/5559, 19%), *Plasmodium vivax* (407/5559, 7.3%), *Plasmodium malariae* (157/5559, 2.8%), and 1 case of *Plasmodium knowlesi*. Most of the cases were male (5343/5559, 96.1%). The complication rates for *P falciparum* and *P ovale* were 11.4% and 3.3%, respectively. Multivariate logistic regression analysis of the relevant factors of malaria complications revealed potential protective factors, including a previous infection by *Plasmodium* (*P*<.001; odds ratio [OR] 0.512, 95% CI 0.422‐0.621), and risk factors, including increased age (*P*=.004; OR 1.014, 95% CI 1.004‐1.024), misdiagnosis at the first clinical visit (*P*<.001; OR 3.553, 95% CI 2.886‐4.375), and the time interval from onset to treatment (*P*=.001; OR 1.026, 95% CI 1.011‐1.042). Subgroup analyses identified risk factors associated with *P falciparum*, which include advanced age (*P*=.004; OR 1.015, 95% CI 1.005‐1.026), initial misdiagnosis during the first clinical visit (*P*<.001; OR 3.549, 95% CI 2.827‐4.455), the time interval from onset to treatment (*P*<.001; OR 1.043, 95% CI 1.022‐1.063), and a delay of more than 3 days from the first treatment to diagnosis (*P*<.001; OR 2.403, 95% CI 1.823‐3.164). Additionally, the risk factors pertaining to *P ovale* involve misdiagnosis at the initial clinical visit (*P*=.01; OR 2.901, 95% CI 1.336‐6.298), the time interval from onset to treatment (*P*=.002; OR 1.095, 95% CI 1.033‐1.160), and the duration from the initial treatment to diagnosis (*P*=.43; OR 1.032, 95% CI 0.953‐1.118). Previous infections can prevent the progression of both *P falciparum* and *P ovale*.

**Conclusions:**

This study showed that the increasing proportion of *P ovale* in recent years should not be ignored. Furthermore, there is a need to improve diagnostic awareness, enhance the capacity of medical institutions, and provide health education for high-risk groups.

## Introduction

Malaria is an infectious disease that continues to pose a significant threat to human health in tropical and subtropical areas [1]. It is listed as 1 of the 3 major infectious diseases by the World Health Organization (WHO), alongside HIV/AIDS and tuberculosis [2]. According to the 2023 World Malaria Report [3] issued by the WHO, malaria remained endemic in 85 countries in 2022 with approximately 249 million cases, leading to 608,000 deaths. The African region has the highest burden of malaria globally, accounting for 93.6% of the global cases in 2022. In fact, the world’s progress toward malaria elimination has stalled since 2017, and the number of cases has rebounded [4]. The incidence of malaria has been on the rise since 2017, notably increasing from 218 million to 233 million cases between 2019 and 2022. The resurgence of malaria cases may pose spillover risks to other nonendemic countries. Consequently, regions without endemic malaria, particularly nations that have recently attained elimination, should remain vigilant against this potential threat and strengthen surveillance of imported malaria [5].

Malaria is one of the most widespread infectious diseases in China, with a long history of endangering people’s physical and mental health. China has implemented a comprehensive set of tailored policies and measures to combat malaria, designed for its specific circumstances [6]. Different departments have collaborated and operated jointly under government leadership to assume responsibility for prevention and control efforts [7]. After 70 years of efforts, China was certified by the WHO as a malaria-free country on June 30, 2021. However, with ongoing globalization and the promotion of the Belt and Road Initiative and China-Africa cooperation strategy, the cross-border flow of people has become more frequent, and overseas sources of infection continue to be imported. Imported malaria poses a significant threat to the consolidation of China's achievements in malaria elimination [8]. Obviously, until malaria is eradicated globally, China still needs to pay attention to the risk of imported malaria, as the introduction of *Plasmodium* parasites may lead to the re-establishment of malaria. This has already been observed in countries where malaria has been successfully eliminated [9-12]. Continuous surveillance in China is used to address the potential impact of imported malaria, a crucial component of the global malaria ecosystem [13]. Before the outbreak of COVID-19, the annual number of imported malaria cases in China was approximately 3000 [14]. The main origin of infection was Africa, with *Plasmodium falciparum* being the predominant species, and with fatal cases due to *P falciparum* occasionally reported [14]. Moreover, dynamic surveillance data have indicated an increased trend of non-*falciparum* malaria infection among the imported malaria cases in China [15-18]. However, previous studies mainly focused on *P falciparum*, and the study on the clinical outcome of *non-falciparum* malaria was neglected [19,20]. Surveillance data in China are supported by reliable clinical diagnosis, which can help to improve the understanding of the field.

This was a retrospective case-based study that used malaria surveillance data from 5 provinces of China from January 1, 2014, to December 31, 2021. Epidemiological characteristics, diagnosis, and treatment were analyzed. Factors influencing complications of malaria cases were explored. The findings provide evidence that will be valuable in optimizing the management strategy of imported malaria cases.

## Methods

### Study Design and Data Sources

The provinces of Anhui, Henan, Hubei, Zhejiang, and Guangxi Zhuang Autonomous Region, China, were selected for this study, according to the epidemics and retransmission risk of imported malaria [21]. Zhejiang is an economically developed province where international communications are frequent, which has a high risk of imported cases [22]. Guangxi is a border province in southern China, where numerous labor workers journey to Africa for gold mining [23]. Historically, malaria was highly prevalent in the provinces of Anhui, Henan, and Hubei [24-26], which means high receptivity, namely, a relatively high risk of re-establishment of malaria. The provinces of Anhui, Hubei, Henan, Zhejiang, and Guangxi (hereinafter referred to as “the five provinces”) were chosen as the study sites.

The data of the imported cases were collected from January 1, 2014, to December 31, 2021, from the 5 provinces using the infectious disease information reporting management system and parasitic disease information reporting management system [21]. We included only cases with singular *Plasmodium* infections and well-documented complications; mixed infections were excluded from the analysis. The collected information included general demographic characteristics, source of infection, the reason for travel, date of onset, first visit unit and time, first visit results, laboratory diagnosis results, types of complications, treatment drugs, and others. Before treating imported malaria cases in the study, blood samples were collected, and the presence of *Plasmodium* species was confirmed by microscope examination and polymerase chain reaction at the provincial reference laboratories, in compliance with national criteria for malaria diagnosis [27].

### Definition of Malaria Complications and Imported Malaria

The definition of malaria complications is that during the course of the disease, other diseases or clinical manifestations induced by malaria were present, but the underlying diseases of the patients were not included. These included brain damage, acute respiratory distress syndrome, shock, hemolysis, severe renal damage, pulmonary edema, severe anemia, acidosis, liver damage, gastrointestinal damage, and others [28].

In this study, the term “imported malaria” refers to individuals who have been diagnosed with malaria parasites in their blood through diagnostic tests, as well as those who have contracted the infection outside of China.

### Statistical Analyses

The case database was established using Microsoft Excel 2021, and IBM SPSS Statistics v26.0 software was used for statistical analyses. The epidemiological characteristics, diagnosis and treatment processes, complications, and other variables of the imported malaria cases were descriptively analyzed. Continuous variables were described as the mean ($\overset{-}{\chi }$) and SD if they were normally distributed, and as medians and IQRs otherwise. Qualitative data are presented as ratios or percentages (%).

Logistic regression was used to identify the factors influencing the occurrence of complications as the dependent variable. The Box-Tidwell method was used to test the linear relationship between the continuous independent variable and the logit (p) of the dependent variable. If the linear relationship was not satisfied, the variable was transformed into a categorical variable. Univariate analysis was used to screen statistically significant independent variables, which were included in the multivariate model for analysis. Further subgroup analysis was carried out according to malaria species (*P falciparum*, *Plasmodium ovale*, *Plasmodium vivax*, *Plasmodium malariae*, and *Plasmodium knowlesi*) to identify the influencing factors of complications of different malaria species. Two-sided tests were used for statistical analysis with *α*=.05.

### Ethical Considerations

This study was approved by the ethical review committee of the National Institute of Parasitic Diseases, Chinese Center for Disease Control and Prevention (No.20190115). Since all data were deidentified from the National Information System for Parasitic Disease Control and Prevention, informed consent was waived. Additionally, the authors obtained permission to access and use the data, ensuring adherence to data usage policies and ethical standards.

## Results

The study received funding in January 2022, and the survey was conducted from June to July 2022. The study included a total of 5559 cases of imported malaria from 5 provinces of China between 2014 and 2021.

### Epidemiological Profile

**Figure 1.**
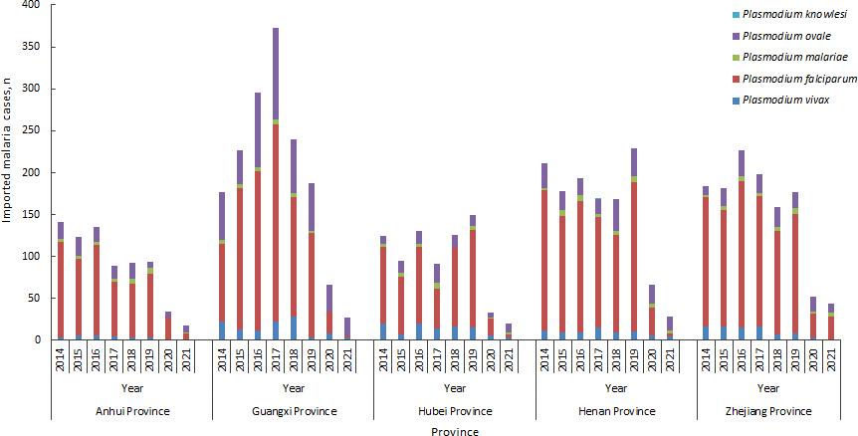
Number of imported malaria cases in 5 provinces of China from 2014 to 2021.

During the study period, a total of 5559 cases of imported malaria were included, including 3940 cases of *P falciparum*, 1054 cases of *P ovale*, 407 cases of *P vivax*, 157 cases of *P malariae*, and 1 case of *P knowlesi*. Most of the cases (5343/5559, 96.1%) were male. The mean age (in years) of the patients was 40 (SD 9.9; range 1‐75). There were 5 children, accounting for 0.1% (5/5559) of the total, who were under 15 years old. The ages of these children were 1, 2, 9, 14, and 15 years. Most of the cases were migrant workers (81.6%, 4536/5559). Of the imported cases, 96.1% (5344/5559) were from Africa. The nations with the highest number of cases were Ghana (843/5559, 15.2%), followed by Nigeria (669/5559, 12%) and Cameroon (551/5559, 9.9%). The full details are provided in Figure 1 and Table 1.

**Table 1.** Characteristics of patients with imported malaria in 5 provinces of China from 2014 to 2021 (N=5559).

Characteristics	Patients
**Gender**	
Male, n (%)	5343 (96.1)
Female, n (%)	216 (3.9)
Age (years), mean (SD)	40 (9.9)
**Species, n (%)**	
*Plasmodium vivax*	407 (7.3)
*Plasmodium falciparum*	3940 (70.9)
*Plasmodium malariae*	157 (2.8)
*Plasmodium ovale*	1054 (19)
*Plasmodium knowlesi*	1 (0)
**Purpose of travel, n (%)**	
Labor	4536 (81.6)
Official duties or business	878 (15.8)
Others	145 (2.6)
**Previous infection** ^**a**^ **, n (%)**	
Yes	3982 (71.6)
No	1577 (28.4)
**Origin of infection** ^**b**^ **, n (%)**	
Africa	5344 (96.1)
Oceania	13 (0.2)
Asia	194 (3.5)
South America	8 (0.1)

a Previous infection: People who have been infected with *Plasmodium*, regardless of the species.

b Origin of infection: One case was Turk. Turkey is a trans-Eurasian country. It is included in Asia because Europe is not malaria-endemic.

### Diagnosis and Treatment

The majority (3582/5559, 64.4%) of the imported malaria cases initially visited medical institutions at or below the county level. Standard treatments were almost always provided (5467/5559, 98.3%). However, 25.6% (1421/5559) of the imported malaria cases did not receive standard treatments on their first visit. Complications occurred in 9% (500/5559) of the cases, as detailed in Table 2.

**Table 2.** Diagnosis and treatment of patients with imported malaria in 5 provinces of China from 2014 to 2021 (N=5559).

Variables		Patients
**Misdiagnosis at the first visit, n (%)**		
	Yes	1421 (25.6)
	No	4138 (74.4)
**Standard treatment^a^** **, n (%)**		
	Yes	5467 (98.3)
	No	92 (1.7)
**The level of the first medical visit unit, n (%)**		
	Country hospital and below	3582 (64.4)
	Municipal hospital	1260 (22.7)
	Provincial hospital	588 (10.6)
	Others	129 (2.3)
**Antimalarial drugs, n (%)**		
	Chloroquine plus primaquine	311 (5.6)
	Artemisinin-based combination therapy	1697 (30.5)
	Injectable artemisinin^b^	1388 (25.0)
	Combination therapy^c^	2072 (37.3)
	Others	91 (1.6)
**Complication**		
	Yes, n (%)	500 (9.0)
	No, n (%)	5059 (91.0)
	Time from symptom onset to first time of treatment (days), median (IQR)	1 (0-3)^d^
	Time from the first time of treatment to diagnosis (days), median (IQR)	0 (0-2)^d^

a Standard treatment: Conform to the standard of use of antimalarial drugs (WS/T 485‐2016).

b Injectable artemisinin: Artemisinin is designed for injection into the body.

c Combination therapy: The treatment of malaria involves the use of two or more medicines.

d A score of 0 was assigned if the patient sought medical attention on the day of symptom onset or was diagnosed on the day of medical attention.

### Distribution of Complications

Complications occurred for all 4 common species of malaria. The most frequently involved protozoan was *P falciparum* (449/3940, 11.4%), followed by *P ovale* (35/1054, 3.3%), *P vivax* (12/407, 2.9%), and *P malariae* (4/157, 2.5%). The proportion of complications of *P falciparum* was the highest, and that of non-*falciparum* malaria was similar (*P*=.84). The 449 cases with complications for *P falciparum* included 68 cases (68/449, 15.1%) with impaired consciousness, 76 cases (76/449, 16.9%) with hepatic damage and 47 cases (47/449, 10.5%) with severe anemia. Four cases of impaired consciousness occurred for both *P ovale* and *P vivax* infections. Full details are provided in Table 3.

**Table 3.** Complications in patients with imported malaria in 5 provinces of China from 2014 to 2021.

Complications	*Plasmodium vivax*, n (%)	*Plasmodium falciparum*, n (%)	*Plasmodium malariae*, n (%)	*Plasmodium ovale*, n (%)
Impaired consciousness	4 (33)	68 (15.1)	0 (0)	4 (11)
Acute respiratory distress syndrome	0 (0)	5 (1.1)	0 (0)	0 (0)
Shock	0 (0)	18 (4)	0 (0)	2 (6)
Hemolysis	0 (0)	12 (2.7)	0 (0)	0 (0)
Renal impairment	0 (0)	4 (0.9)	0 (0)	0 (0)
Pulmonary edema	0 (0)	2 (0.4)	0 (0)	0 (0)
Severe anemia	1 (8)	47 (10.5)	0 (0)	2 (6)
Acidosis	0 (0)	2 (0.4)	0 (0)	0 (0)
Hepatic damage	1 (8)	76 (16.9)	2 (50)	10 (29)
Gastrointestinal damage	2 (17)	37 (8.2)	0 (0)	4 (11)
Others^a^	4 (33)	178 (39.6)	2 (50)	13 (37)
Total	12 (100)	449 (100)	4 (100)	35 (100)

a Others: Other diseases caused by *Plasmodium* do not include underlying diseases.

### Analysis of Factors Influencing Complications

Univariate analysis results showed that a previous history of *Plasmodium* infection was a protective factor, while gender (male), age (years), misdiagnosis at the first visit, time from symptom onset to first time of treatment (days), and time from the first time of treatment to diagnosis (days) were factors associated with complications. Multivariate analysis showed that a previous history of *Plasmodium* infection reduced the risk of complications (odds ratio [OR] 0.512, 95% CI 0.422‐0.621). Factors increasing the risk of complications were age (1.4% increased risk for every year; OR 1.014, 95% CI 1.004‐1.024), misdiagnosis at the first visit (OR 3.553, 95% CI 2.886‐4.375), and delay between the onset of illness and visit (2.6% per day; OR 1.026, 95% CI 1.011‐1.042). The full details are provided in Table 4.

**Table 4.** Logistic regression analyses of complications of patients with imported malaria in 5 provinces of China from 2014 to 2021.

Category	Crude		Adjusted	
	*P* value	Odds ratio (95% CI)	*P* value	Odds ratio (95% CI)
Gender^a^	.04	0.651 (0.433-0.979)	.01	0.667 (0.436-1.021)
Age (years)	.01	1.012 (1.003-1.022)	.004	1.014 (1.004-1.024)
Misdiagnosis at the first visit	<.001	4.023 (3.334-4.854)	<.001	3.553 (2.886-4.375)
Previous infection^b^	<.001	0.418 (0.347-0.504)	<.001	0.512 (0.422-0.621)
Time from symptom onset to first time of treatment (days)	.02	1.017 (1.002-1.032)	.001	1.026 (1.011-1.042)
Time from the first time of treatment to diagnosis (days)	<.001	1.065 (1.047-1.084)	.15	1.015 (0.995-1.035)

a Gender: Female patients used as controls.

b Previous infection: People who have ever been infected with *Plasmodium*, regardless of the species.

Subgroup analyses were performed to identify the influential factors for *P falciparum* and *P ovale*. Due to an insufficient sample size for complications, *P vivax*, *P malariae*, and *P knowlesi* were not included. The univariate analysis results showed that the factors influencing complications of *P ovale* were previous infection, misdiagnosis at the first visit, time from symptom onset to first time of treatment (days), and time from the first time of treatment to diagnosis (days). The influencing factors of *P falciparum* included previous infection, age (years), misdiagnosis at the first visit, time from symptom onset to the first time of treatment (days), and time from the first time of treatment to diagnosis exceeding 3 days.

Multiple logistic regression analysis results revealed that previous infection decreased the risk of complications of *P ovale* (OR 0.444, 95% CI 0.199‐0.990) and *P falciparum* (OR 0.597, 95% CI 0.485‐0.735). Misdiagnosis at the first visit increased the risk of complications of *P ovale* (OR 2.901, 95% CI 1.336‐6.298) and *P falciparum* (OR 3.549, 95% CI 2.827‐4.455), as did each day in the time from symptom onset to the first time of treatment, with a 9.5% daily increase for *P ovale* (OR 1.095, 95% CI 1.033‐1.160) and 4.3% for *P falciparum* (OR 1.043, 95% CI 1.022‐1.063). Age was also a risk factor for increased risk of complications for *P falciparum*, with a 1.5% yearly increase (OR 1.015, 95% CI 1.005‐1.026). Finally, the time from the first time of treatment to diagnosis exceeding 3 days increases the risk of complications of *P falciparum* (OR 2.403, 95% CI 1.823‐3.164). The full details are provided in Table 5.

**Table 5.** Logistic regression analyses of risk factors for complications of different parasite species of imported malaria in 5 provinces of China from 2014 to 2021.

Variables		Crude		Adjusted	
		*P* value	Odds ratio (95% CI)	*P* value	Odds ratio (95% CI)
***Plasmodium falciparum***					
	Misdiagnosis at the first visit	<.001	4.410 (3.601-5.400)	<.001	3.549 (2.827-4.455)
	Age (years)	.01	1.013 (1.003-1.023)	.004	1.015 (1.005-1.026)
	Previous infection	<.001	0.483 (0.396-0.590)	<.001	0.597 (0.485-0.735)
	Time from symptom onset to first-time of treatment (days)	.004	1.029 (1.009-1.048)	<.001	1.043 (1.022-1.063)
	Time from the first time of treatment to diagnosis exceeding 3 days	<.001	4.889 (3.893-6.141)	<.001	2.403 (1.823-3.164)
***Plasmodium ovale***					
	Misdiagnosis at the first visit	.001	3.168 (1.607-6.246)	.01	2.901 (1.336-6.298)
	Previous infection	.02	0.382 (0.175-0.835)	.047	0.444 (0.199-0.990)
	Time from symptom onset to first time of treatment (days)	.01	1.077 (1.017-1.139)	.002	1.095 (1.033-1.160)
	Time from the first time of treatment to diagnosis (days)	.02	1.075 (1.013-1.141)	.43	1.032 (0.953-1.118)

## Discussion

### Principal Findings

The prevalence of *P falciparum* still predominates, while the proportion of *P ovale* has increased among imported malaria cases in China. This trend may indicate the underestimation of *P ovale* malaria in Africa and nonstandardized treatments in at-risk populations. Additionally, our findings indicate that *P ovale* can lead to severe complications, including brain damage. Health care providers in China should continually enhance their awareness and diagnostic abilities pertaining to imported malaria in order to improve patient outcomes.

According to the dynamic surveillance data, a total of 5559 cases of imported malaria were included in the 5 provinces during this study period, 3940 (70.9%) cases involving *P falciparum* and 1054 (19%) cases involving *P ovale*. The number of *P falciparum* cases still ranks first, and the proportion of *P ovale* cases has increased, consistent with the data-related domestic studies in recent years [15,16]. In contrast, the monitoring data from Italy from 2011 to 2017 indicated that the proportion of imported *P ovale* is only approximately 5% (range 3%‐8%), and *P falciparum* was the most frequent species identified, with 78% (range 69%‐84%) of infections over the study period [29]. The surveillance of all imported malaria cases in Bulgaria from 2000 to 2020 also showed that *P falciparum* accounted for the highest proportion (64.65%), followed by *P vivax* (28.45%) and *P ovale* (3.45%) [30]. Furthermore, a study from the European surveillance reported that *P falciparum* accounted for the majority of those cases (n=28,070, 89%). *P ovale*, *P malariae,* and *P vivax* represented 6%, 3%, and 2% of the infections, respectively [31]. The increased prevalence of *P ovale* among imported malaria in China may reflect the underestimated prevalence of *P ovale* malaria in Africa, unstandardized treatments, using more sensitive and accurate nucleic acid detection methods, and other factors. Further studies are needed to explore the underlying causes of the changes in the epidemic situation of malaria. These data will help inform the optimizations and adjustments of control measures for imported malaria in China.

This study scrutinized the complications of malaria and the influential factors. Among the 5559 imported malaria cases, 500 (9%) complications were observed. Complications were reported for all 4 species. The protozoan that was most frequently associated was *P falciparum*, with similar rates for non-*falciparum* malaria. The comparative data reveal that the situation of *P falciparum* is more complicated and serious. Four cases of brain damage were reported for both *P vivax* and *P ovale*. This is the same as reported elsewhere [32,33]: both *P vivax* and *P ovale* can lead to severe cases, can induce adverse clinical outcomes, and can be lethal. Thus, further observational studies on the clinical characteristics of non-*falciparum* and standardized treatments are needed to provide new evidence for the management of imported malaria cases.

Multivariate logistic regression analysis showed that previous infection by *Plasmodium* offered protection against the complications of imported malaria. Patients who had previously been infected with *Plasmodium* may have acquired partial immunity, which decreases the risk of complications [34]. However, it depends on the factor of time as the level of acquired immunity decreases over time. In addition, previous infection by *Plasmodium* can increase a person’s awareness of malaria-related symptoms. When patients exhibit symptoms resembling a *Plasmodium* infection, they are more likely to consider the possibility of having *Plasmodium* if they have been previously infected. This may instill a strong awareness of active medical treatment and timely and effective treatments [35]. Contrary to previous infection by *Plasmodium*, age is a risk factor for complications. With increasing age, the body’s various functions and resistance decline [36], which increases the possibility of complications. However, this may also be due to the fact that the imported malaria cases mainly concern young and middle-aged people.

The findings of this study also emphasize the importance of initial diagnosis. If the diagnosis is not correct at the first visit, the patient may not be treated in time. The resulting delay may result in various complications. The times from symptom onset to the first time of treatment and from the first time of treatment to diagnosis are important risk factors for complications, which are related to the prolonged course of the disease. The current findings echo the serious consequences of delay in medical care seeking and diagnosis of malaria cases that have been previously reported [37,38]. Further subgroup analysis showed that the prolonged time from onset to first time of treatment was a risk factor for the complications of *P ovale*, while no statistical association was found between the time from the first time of treatment to diagnosis and the complications of *P ovale*. This may be due to the low density of parasites in the blood of the infected individual and the relatively mild clinical symptoms [28,39]. Therefore, patients often fail to seek timely medical treatment after the onset of the disease, resulting in a prolonged interval between symptom onset and medical treatments. However, for *P falciparum*, both the prolonged time from symptom onset to the first time of treatment and from the first time of treatment to diagnosis are risk factors for complications. This is due to the rapid clinical progress of *P falciparum*, which repeatedly replicates over the course of 48 h inside erythrocytes, resulting in exponential growth and rapid disease progression [40,41]; the delay from symptom onset to diagnosis has an important impact on the development of the disease.

### Study Limitations

There are several limitations to this study. The data were obtained from a routine surveillance system, and the study was retrospective. The possibility of recall bias cannot be discounted. Moreover, the complications of malaria are complex, and different epidemiological investigators may not record all clinical complications. Furthermore, it is important to note that this study did not encompass border areas, so the findings may not be directly applicable to those regions. In border areas, the prevention and control of malaria rely more on effective information communication [42].

### Conclusions

While *P falciparum* represents the highest proportion among the 5 malaria species and exhibits a higher fatality rate in comparison to the other species [43], it is crucial to acknowledge the importance of the other malaria species. In recent years, the proportion of imported *P ovale* has been on the rise, and serious complications can endanger patient survival. Diagnostic awareness and the capacity of medical institutions must be improved. The present findings emphasize the importance of initial diagnosis. Strengthening the team of professionals is crucial [44]. In addition, the challenge of complications associated with imported malaria indicates the need for health education for risk groups.

## Abbreviations

OR: odds ratio
WHO: World Health Organization
